# Implementation salvage experiences from the Melbourne diabetes prevention study

**DOI:** 10.1186/1471-2458-12-806

**Published:** 2012-09-19

**Authors:** James Dunbar, Andrea Hernan, Edward Janus, Nathalie Davis-Lameloise, Dino Asproloupos, Sharleen O’Reilly, Amy Timoshanko, Elizabeth Stewart, Catherine M Bennett, Greg Johnson, Rob Carter

**Affiliations:** 1 Greater Green Triangle University Department of Rural Health, Flinders University and Deakin University, Warrnambool, VIC, 3280, Australia; 2 Department of Medicine, North West Academic Centre, The University of Melbourne, Western Hospital, Footscray, VIC, 3011, Australia; 3 Faculty of Health, Deakin University, 221 Burwood Highway, Burwood, VIC, 3125, Australia; 4 Diabetes Australia – Victoria, 570 Elizabeth Street, Melbourne, VIC, 3000, Australia; 5 Deakin Health Economics, Deakin Strategic Research Centre - Population Health, Faculty of Health, Deakin University, 221 Burwood Highway, Burwood, VIC, 3125, Australia

**Keywords:** Implementation, Salvage strategy, Type 2 diabetes, Prevention, Effectiveness, Randomised controlled trial

## Abstract

**Background:**

Many public health interventions based on apparently sound evidence from randomised controlled trials encounter difficulties when being scaled up within health systems. Even under the best of circumstances, implementation is exceedingly difficult. In this paper we will describe the implementation salvage experiences from the Melbourne Diabetes Prevention Study, which is a randomised controlled trial of the effectiveness and cost-effectiveness nested in the state-wide *Life! Taking Action on Diabetes* program in Victoria, Australia.

**Discussion:**

The Melbourne Diabetes Prevention Study sits within an evolving larger scale implementation project, the Life! program. Changes that occurred during the roll-out of that program had a direct impact on the process of conducting this trial. The issues and methods of recovery the study team encountered were conceptualised using an implementation salvage strategies framework. The specific issues the study team came across included continuity of the state funding for Life! program and structural changes to the Life! program which consisted of adjustments to eligibility criteria, referral processes, structure and content, as well as alternative program delivery for different population groups. Staff turnover, recruitment problems, setting and venue concerns, availability of potential participants and participant characteristics were also identified as evaluation roadblocks. Each issue and corresponding salvage strategy is presented.

**Summary:**

The experiences of conducting such a novel trial as the preliminary Melbourne Diabetes Prevention Study have been invaluable. The lessons learnt and knowledge gained will inform the future execution of this trial in the coming years. We anticipate that these results will also be beneficial to other researchers conducting similar trials in the public health field. We recommend that researchers openly share their experiences, barriers and challenges when conducting randomised controlled trials and implementation research. We encourage them to describe the factors that may have inhibited or enhanced the desired outcomes so that the academic community can learn and expand the research foundation of implementation salvage.

## Background

Traditionally randomised controlled trials (RCTs) are scientific experiments that utilise controlled environments to allocate a treatment to the intervention group, while the control group receives a placebo or no treatment at all [1]. RCTs are considered to be the most reliable method for gathering evidence about the efficacy of public health interventions, as they uphold high standards of internal validity inherent in their methodological design [1-3]. Internal validity is defined as the extent to which the independent variable (health intervention; drug trial or lifestyle changes program) produces the desired effect or outcome [4]. Although RCTs provide reliable evidence about the efficacy of an intervention, they lack the ability to examine external validity which ascertains whether the results or effects seen in a small population can be expanded to or generalised to other populations or settings [4]. External validity is a measure of the effectiveness of an intervention in the real world [2].

The primary reason for conducting RCTs of public health interventions is to translate them into scaled up, system-wide programs [5]. A missing link between science and practice called implementation science or research has emerged through studying the process of implementing evidence-based programs and practices in the real world [6-8]. Implementation research is the study of methods to promote the systematic uptake of research findings and other evidence-based practices into routine practice, to improve the quality and effectiveness of health services [9]. It includes the study of influences on healthcare professionals and organisational behaviour including processes, barriers and facilitators [9].

Many policies based on apparently sound evidence from RCTs encounter difficulties when being scaled up within health systems. Even under the best circumstances, implementation is exceedingly difficult. The road is likely to be full of unexpected detours, blind alleys, new opportunities and changing constraints. These constraints may vary over time leading to a trial and error approach to discover feasible solutions [10].

Not as well discussed in the literature is the process of evaluating an RCT alongside an already established and operational health intervention or program occurring in a real world, primary or community health care setting. We did not find any evidence in the literature of this particular evaluation methodology or the specific problems or issues that may be encountered when embarking on such an evaluation.

Implementation Salvage Strategies is a framework proposed by Hoagwood et al. (2011) to describe and understand the possible factors that can influence the conduct of a trial within real world circumstances and the possible solutions to such problems [11]. This model highlights specific factors that can impede on different phases of a trial, and is used to analyse, describe and promote strategies to rescue a study where it otherwise might be terminated.

In this paper we will describe the implementation salvage experiences for the Melbourne Diabetes Prevention Study (MDPS), which is an RCT of the effectiveness and cost-effectiveness nested within the *Life! Taking Action on Diabetes* program (Life!) conducted in Victoria, Australia. This state-wide type 2 diabetes prevention program for high risk individuals is funded by the Victorian Government Department of Health Victoria and delivered by Diabetes Australia – Victoria (DA–Vic), a non-government organisation (NGO), peak consumer body and leading charity representing all people affected by diabetes and those at risk.

We now describe how the need for adjustments to the real world Life! program created challenges for the nested RCT and our implementation salvage for the MDPS.

## Context

### Diabetes prevention

It was estimated that approximately 366 million adults, 8.3% of the adult population worldwide, had diabetes in 2011. This is expected to increase to 552 million adults, 9.9% of the expected population worldwide by 2030 [12]. These alarming statistics provide evidence for the need to prevent type 2 diabetes (T2D). Several recent clinical trials have demonstrated that lifestyle modifications with weight loss combined with dietary change and moderate exercise can reduce the incidence of T2D by up to 58% for people at high risk [13-15]. Indeed, these have been shown to be even more effective than drug treatment in clinical trials, and have a prolonged impact [13,16,17]. The challenge has been to translate these findings into the real world.

Encouraging results from small efficacy evaluative trials conducted in the US, Finland and our own Greater Green Triangle Diabetes Prevention Program (GGT DPP) in Australia, have contributed to an emerging evidence base in this field [18-20]. While there have been some positive results and lessons learnt about preventing diabetes in the real world, there were limitations in these studies. The samples were in some cases self-selected, were often small, some lacking a formal comparison group (control), and follow-up was generally short with the intervention remaining relatively intensive [21]. Understanding how to implement interventions on a large-scale is the next step in the process of translating diabetes prevention into multiple and multi-level population strategies that span the state or national stage [22]. There have been reported accounts in the literature of the challenges of translating clinical trials into effective population programmes, along with the development of recommendations and guidelines for prevention of type 2 diabetes [23,24]. These frameworks are useful for evaluation and implementation processes. In addition more research is required to understand the effectiveness of interventions, their acceptability, uptake, reach, cost and how they work in different population sub-groups and via which mechanisms. This is particularly important for public health research, practice and policy [21]. Evaluations of this nature can determine program intensity, fidelity and sustainability in the long term.

Finland was the first country to undertake a large-scale diabetes prevention intervention. The national Development Programme for the Prevention and Care of Diabetes (DEHKO 2000–2010) was the first of its kind aiming to improve the quality of diabetes care, the support for self-care and the prevention of T2D and its complications [25]. One of the three main goals was to develop a community-based high risk program in primary and occupational care to prevent T2D, called the National Type 2 Diabetes Prevention Programme in Finland: FIN-D2D [26]. Other large scale diabetes prevention projects include the Diabetes in Europe – Prevention using Lifestyle, Physical Activity and Nutritional Intervention (DE-PLAN study) across Europe [27], and the Life! Taking Action on Diabetes program (Life!) in the Australian state of Victoria [28,29].

### Life! taking action on diabetes program

Life! is a state-wide, group based, lifestyle change program with the aim of targeting Victorians aged 50 years or over at high risk of developing T2D. The goals of the program are based on modifications to diet and physical activity. The program is conducted by Life! facilitators who are certified health professionals. Life! has direct lineage from the Finnish Diabetes Prevention Study (DPS) [15], the Good Ageing in Lahti region (GOAL) Implementation trial [18] and is the scaled-up version of the GGT DPP [20]. In addition, the Victorian Government’s Healthy Living Course randomised controlled trial in 2005 examined the feasibility of a six session group-based lifestyle intervention in a Victorian population [30], which also informed the development of the Life! program. The Life! program optimises intervention fidelity through a rigorous annual training and accreditation program for facilitators [31].

### The Melbourne diabetes prevention study (MDPS)

The Melbourne Diabetes Prevention Study (MDPS) is a research project set up to study in detail a cohort of individuals undertaking the Life! program in order to evaluate the effectiveness of a large-scale prevention program, and importantly the cost-effectiveness. There is a paucity of data to assess the long-term clinical and economic impact of diabetes prevention programs. While there have been some modelling studies that have determined that lifestyle intervention is cost-effective for those with impaired glucose regulation [32,33], uncertainties exist about model parameters, real costs and benefits of screening, and practical considerations about the affordability, acceptability and feasibility of interventions [21,32-34].

The MDPS is an RCT which aims to evaluate the efficacy and effectiveness of Life! by monitoring participants’ clinical and behavioural outcomes before and after the Life! intervention, as previously reported [35]. An economic assessment of Life! is also included in the MDPS to determine its ‘value-for-money’. The MDPS also provides the opportunity to utilise implementation research methodology to examine the process of implementing an evidence-based diabetes prevention program in the real world.

In brief, the MDPS intends to recruit 796 participants for this open randomised clinical trial, 398 will be allocated to the intervention arm and 398 to the usual care arm. Several methods of recruitment will be used in order to maximise the number of participants. Individuals aged 50 to 75 years will be screened with a risk tool (AUSDRISK[36]) to detect those at high risk of developing T2D. Those with existing diabetes will be excluded. Intervention participants will undergo anthropometric and laboratory tests, and comprehensive surveys at baseline, at three months (following the main part of the intervention) and 12 months, while control participants will undergo testing at baseline and 12 months only.

The intervention consists of an initial individual session followed by a series of five structured-group sessions. The first four group sessions will be carried out at two week intervals and the fifth session will occur eight months after the first group session. The intervention is based on the Health Action Process Approach (HAPA) model [37-39] and sessions empower and enable participants to follow the five goals of the Life! program. The intervention fidelity is maintained through a thorough accreditation and training process for facilitators as outlined above. It is also achieved through employing a single facilitator to run all of the MDPS groups. This minimises the variability in course delivery and content participants will receive.

The primary outcomes under investigation are changes in diabetes and cardiovascular disease (CVD) risk as determined by changes in weight, waist circumference, fasting plasma and 2 h glucose, blood pressure and lipids at the baseline, 3 and 12 month assessments. The reduction in diabetes risk is calculated from the reduction in weight and waist circumference and the reduction in cardiovascular risk from changes to the Framingham risk score which incorporates the changes in the individual risk factors. Secondary outcomes consist of changes in psychosocial and quality of life measurements.

## Discussion

### Evaluation roadblocks

Encountering unexpected problems while conducting clinical trials is an inherent part of research, as discussed in detail by many authors [1,40-45]. Translating evidence-based practices and process from RCTs to large-scale implementation projects can be an even bigger test, and evaluation of the effectiveness of such projects can be an enormous task as public health interventions are often complex and difficult to evaluate. This evaluation is complicated in the MDPS as it is an RCT being conducted within an already established and operational, large-scale health intervention program.

The MDPS intervention arm sits within an evolving larger scale implementation project, the Life! program. Changes that occurred in the roll-out of that program had a direct impact on the process of conducting this trial. Life! is an evolving and changing program that is subject to modifications made at a political and environmental level beyond the control of the MDPS researchers. The nature of the Life! intervention was changing over time. This custom-tailoring of the Life! program was essential for its progress and success, and to assist with community engagement, promoting risk assessment, program entry, and sustainability. Such changes have implications on the fidelity and usefulness of the MDPS.

Life! was initiated in 2007, with the MDPS starting approximately 15 months later. From inception of the Life! program until 2010 numerous adjustments were made to the eligibility criteria, referral process, structure and content, as well as alternative program delivery for different population groups. These adjustments are outlined in detail below.

Ethics approval for the MDPS was obtained from Deakin University Human Research Ethics Committee (Project Code 2009–066).

### Lessons learnt from MDPS

It was decided by the research team in 2009–10 to pause the MDPS after a total of n = 92 individuals completed the study and then to decide how to proceed further. This initial phase we now refer to as the preliminary MDPS study (pMDPS) [35]. This situation arose due to difficulties with recruiting participants into the RCT and due to uncertainties about what version of the Life! intervention was actually being evaluated. This break has given the study team the opportunity to reflect upon the study, to process the available information, to report preliminary results [35], and to learn from the experiences of undertaking implementation research in this ever changing context.

We have now started again and the new study called MDPS is informed by the pMDPS experience. As there were changes to the intervention and other things, the pMDPS and MDPS and their participants are being treated as separate entities to avoid threats to internal validity in both studies.

The summary in Table 1, with a *few* key examples explained in more detail below, outlines the issues the study team encountered in the pMDPS and methods of recovery that were and will be employed in the future execution of the MDPS. Implementation salvage strategies and techniques as discussed by Hoagwood et al. (2011) were used as a basis for conceptualising the issues and solutions outlined below [11].

**Table 1:** Melbourne diabetes prevention study evaluation roadblocks and salvage strategies

**MDPS evaluation roadblock**	** Threat to trial validity**	** Brief explanation**	** Salvage strategy**
**Staff**	Logistical implementation issue	High turnover of staff in the initial stages of conducting the trial led to lack of communication, inconsistency in trial management, inadequate resource allocation, and time delays to recruit participants.	· A strategic leadership and management role was created to oversee the trial and provide an essential link between external stakeholders and the study team.
			· Effort and time was allocated to identify and employ a study coordinator, who wasexperienced in research coordination with a local knowledge of health care organisations/referral pathways/the Life! Program and who could successfully manage the trial.
**Sample Selection**	Under-recruitment and contamination of control group	Recruitment for MDPS had to replicate what was happening in the real world. Life! recruitment strategies included referral sources such as the community. This took away resources and time from establishing relationships and partnerships with local general practice clinics and divisions.	· MDPS reallocated resources and effort to source casual ‘recruiting’ staff and mobilize them to develop a presence at local community organisations such as health clubs/gymnasiums, pharmacies, local churches, University of the Third Age campuses and community fetes and expos.
		Additionally, earlier than expected state wide roll out of Life! meant that MDPS had to compete with Life! providers for participants.	· The study team closely monitors recruitment activities and adapts and refines the recruitment processes at regular intervals.
**Setting**	Logistical and procedural implementation issue	In order to conduct the trial research staff needed a venue to perform clinical tests and the group sessions. Venue hire was expensive and general practice and division staff, and Life! Providers were not directly involved in the trial.	· The study team engaged front line agencies (community venues and Life! providers) through establishing a partnership agreement that outlines the benefits for each party and the roles and responsibilities expected throughout the duration of the trial (including clear referral pathways to refer patients to MDPS and provision of venues).
**Recruitment**	Under-recruitment and under-estimation of effectiveness,	After investigating the recruitment profile of the regions targeted in this study it was recognized that there is a tight and not necessarily readily accessible pool of potential participants over the age of 50 at high risk of diabetes (approximately 8,000 people [60,61]). Therefore recruitment procedures need to efficiently capture and engage the population at risk.	· In addition to expanding recruitment sources from the community, recruitment was also extended to community pharmacies in the local area.
			· Approaches to creating and maintain effective collaborations and partnerships can be rolled out to other divisions of general practices or community health services throughout eastern metropolitan Melbourne, as a method to increase the catchment area of potential study participants.
**Participant characteristics**	Selection bias	Many factors influence recruitment at the participant- level. These factors include low perception of diabetes risk [52] and low motivation to participate in trials.	· Risk awareness increased through social media and marketing from DA-Vic at a local level to promote the Life! program.
			· Promotional materials have been developed for the study to engage health professionals, organisations and the general public. These materials have utilised local area facts to enhance personalisation of the program to potential participants of the trial.
**Continuity of State funding for Life! program**	Logistical implementation issue	Life! was dependent on the State health department’s continued funding beyond the period 2007–11. This funding was not automatically guaranteed. Therefore MDPS was reliant on this funding as well, which caused study delays whilst waiting to receive confirmation about ongoing funding.	Although in May 2011 Life! was granted further funding for another four years, the MDPS research team developed contingency plans during this time of uncertainty that consisted of:
			· Strengthening already established relationships with partner organisation (DA-Vic) to mobilise strategic advocacy for the program.
			· Proposing to self-fund or source alternative funding to run the intervention for trial participants.
**Structural changes to Life!**	Intervention validity	Life! is a real world program that continually changes and evolves through time. This made it difficult to accurately assess what was being implemented in the real world and therefore difficult to evaluate the intervention effect.	· To maintain fidelity of the intervention, the MDPS research team improved communication with DA-Vic to have more input over what was being delivered to trial participants. This included a partnership agreement between local Life! providers who would run a specified modified version of the Life! program for the forthcoming MDPS.

### Implementation salvage strategies

#### Issue: sample selection

Two major challenges of conducting an RCT are recruitment and retention of participants. Inadequate recruitment is deleterious to RCTs, with consequences being reduction of power to detect intervention effects if target numbers are not achieved, and/or protracted recruitment. Delays may result which affect the generalisability of findings, and increase costs due to extension of time, which can all lead to premature termination of the trial [40,43,45]. Puffer and Torgerson (2003) state that in a survey of studies conducted from 2000–2001 approximately 60% of clinical trials had failed to meet their intended recruitment target or required time extensions to do so [46]. Furthermore, evidence suggests that response rates can be as low as 1% in intervention studies [41]. The success of clinical research is dependent on recruitment and retention of study participants.

As the MDPS aim is to evaluate the effectiveness and cost-effectiveness of the Life! program, recruitment strategies had to be concurrent with what was happening in the real world. In addition to GP recruitment, program referral occurred through accredited Life! Facilitators, the local community, partner agencies, the general public and the workplace. The political and environmental context influenced recruitment strategies. One was Victorian state-wide roll out of Life! which impacted on specific areas of metropolitan Melbourne that were originally reserved for MDPS recruitment only. This left the MDPS to compete within its allocated recruitment area with the Life! program. It is probable that when competing side by side like this, potential participants would prefer to enrol in the Life! program as they are guaranteed to receive the intervention [47].

General Practitioners (GPs) were required to confirm eligibility for the Life! program through completion of a referral form that included biochemical and anthropometric measures. Newly diagnosed T2D was a specific exclusion criterion. During this process GPs may have experienced some degree of ‘clinical equipoise’ [48] whereby their preference was to enrol participants into the Life! program over the MDPS as they wanted their patients to receive and benefit from the intervention immediately, believing that it would be ethically unjust not to do so. Clinical equipoise has been demonstrated to have a detrimental effect on recruitment [49]. When clinicians are uncertain about a treatment or they believe one treatment is more favourable than another, it can result in them not referring patients into trials where the ‘unfavoured or uncertain’ treatment is being studied [48].

#### Salvage strategy

MDPS salvage strategies have centred on establishing a systematic process for monitoring the success of recruitment streams at regular intervals and adapting recruitment activities accordingly. While the preliminary MDPS demonstrated that direct community recruitment yielded the highest numbers of recruits [50], a more targeted approach to recruitment in community and pharmacies has refined this process and reduced the financial burden to the study. The study team has broadened the geographic area for recruitment, and has utilised health professionals (such as Pharmacists) with current links to health care agencies in these areas to act as recruiters for the study. In addition, the study team has invested considerable time and resources into enhancing GP recruitment through educational seminars outlining the benefits of the MDPS, the newly revised content and structure, and differences between MDPS and the Life! program in order to prevent further clinical equipoise from occurring.

Potential contamination of control participants by them receiving the intervention (Life! program) is being monitored and will be taken into account during the analysis. Further analysis may also be undertaken to determine if there are any effects of selection bias. This will be done by comparisons between recruitment areas and between groups with different patient characteristics (such as motivation or perceived risk of diabetes) to see if there are any substantial differences.

#### Issue: recruitment

The MDPS utilised messages promoting free clinical tests. Our observations indicated that this may not have been an effective incentive to attract participants. This is in contrast to literature from the US that suggests that free health checks are an incentive to participate in clinical trials [19,51]. This could be true in the US context where health care is not free or is otherwise heavily subsidized for most US citizens, unlike the universal health care coverage provided in Australia.

Also the perceived importance of diabetes prevention by the public is low. Berryman et al. (2009) have shown that diabetes is not regarded as an issue of great concern compared with other diseases, that perceived personal risk of contracting diabetes is low, and the seriousness of diabetes is underestimated because management appears straightforward [52].

Changing attitudes towards diabetes and participation in its prevention is difficult. Motivating individuals to attend a diabetes prevention program and change lifestyle behaviours requires more than an advertising message. Recruitment strategies used in RCTs require personalisation of the risk message by a suitable health professional as they are a trusted source of information and advice [52], but GP involvement is time-consuming and impractical in some contexts [47,53]. Additionally, GPs own beliefs relating to the efficacy, effectiveness and side-effects of a program have been shown to influence both doctor and patient screening and recruitment in clinical trials [47,48].

#### Salvage strategy

Although many of the participant characteristics that influence recruitment into clinical trials were outside of the control of the study investigators, there are some ways to overcome these challenges. Reallocating resources and effort to source ‘recruiting’ staff and mobilise them to develop a presence at local community organisations such as health clubs/gymnasiums, local churches, University of the Third Age campuses and community fetes and expos is a method to try and increase familiarity, trust and knowledge of the study with community members. Recruitment was also extended to community pharmacies in the local area. Strengthening relationships with potential participants is a method to reduce the barriers participants encounter when receiving health advice from a non-trusted source.

It has been demonstrated that strategies focusing on increasing potential participants’ awareness of the health problem, its impact on health, and their engagement in the learning process may increase recruitment into trials [40]. These points will be taken into account when tailoring how recruiters approach and explain the study to potential participants in the community. The use of AUSDRISK as a screening tool is an effective way to personalise risk of T2D at an individual level, as demonstrated in the GGT DPP efficacy trial [54]. The introduction of the new individual session for MDPS anticipated in future for the Life! program should overcome some of these difficulties.

#### Issue: structural changes to Life!

The Life! facilitator and participant workbooks were adapted from the course content delivered in the GGT DPP [20], GOAL Implementation Trial [18] and Finnish DPS [15]. Originally the course consisted of six group sessions that offered participants skills, knowledge, support and advice needed to make lifestyle changes in order to prevent T2D. The content of the course sessions were developed using health psychology theories of health behaviour change [37-39,55,56] and the Health Action Process Approach (HAPA) [38,57]. A trained facilitator [58] conducted each of the six sessions, the first five over three months and the final session at eight months. A physiotherapist/exercise physiologist and dietitian co-facilitated sessions three and four related to physical activity and nutrition respectively.

In early 2010, new participant manuals were introduced to accommodate individuals from various culturally and linguistically diverse backgrounds and those with low literacy levels. This new version included use of more illustrations to convey messages and supplementary materials for specific cultural needs. Additionally a more ‘streamlined’ approach was undertaken with regards to the structure of the sessions, with some changes made to content appearance and timing, to avoid repetition of curriculum. The dietitian and a physiotherapist/exercise physiologist changed to co-facilitating sessions two and four. This modification was made in response to requests from participants to have dietary information appear earlier in the course.

Originally individuals at high risk of developing T2D aged 50 years or over were eligible for Life! High risk was defined as scoring 15 or over on the AUSDRISK tool [36]. Those of Aboriginal or Torres Strait Islander (ATSI) descent, aged 18 years and over with an AUDRISK score of 12 more were also eligible.

Eligibility criteria were revised in July 2010 to include individuals aged 50 years or over at high risk of T2D, who score 12 or more on the AUSDRISK, and additionally, any individual over the age of 18, regardless of AUSDRISK score, who were of ATSI descent or had previously been diagnosed with either gestational diabetes mellitus or cardiovascular disease (specifically ischemic heart disease). In addition, individuals who had received an occupational health assessment [59], and had an AUSDRISK score of 12 or more and aged 18–39 were funded to participate in the Life! program from a separate source.

#### Salvage strategy

Due to the numerous adjustments made to the eligibility criteria, referral processes, structure and content as described above, it was decided to make a small number of modifications to the MDPS intervention, to ensure consistency between the number of sessions offered to the future MDPS participants, and the content within those sessions. When re-started, MDPS will examine the version of Life! that we anticipate will be implemented in 2012.

One of the major changes to the MDPS version of Life! is that instead of running a six group-session program, the MDPS version of Life! will be modified to consist of an individual session at the beginning of the intervention phase and five group sessions. This parallels the Life! Program, which has a separately funded ‘first visit’. Participants will still receive the intervention from Facilitators employed or contracted through accredited Life! providers using the current Life! materials and manuals. It will be delivered using a standardised approach for all MDPS participants. To achieve this, required partnership agreements between local service providers of the Life! program and Life! facilitators were created, and they will run the new agreed version of MDPS. Approval for this was given by DA-Vic. It is hoped that this approach can be rolled out as a successful model to efficiently and systematically create partnerships with other sites who may be involved in the trial, therefore becoming a multi-site trial.

Although there were these changes to the Life! eligibility criteria, the MDPS research team decided to stay with the original criteria of adults aged 50 years or over with an AUSDRISK score of 15 or more, as this was what the funding body had endorsed and would contribute information on the cost-effectiveness of alternate design options.

### Discussion of salvage strategies

#### What roadblocks were identified before starting the MDPS?

Although the MDPS research team did not perform a formal risk analysis, some potential roadblocks were identified when designing the MDPS. These included issues such as recruitment and dropout rates, and the impact of these were based on previous experience of implementing DPPs. Also, the issues surrounding usual care and other potential government or community diabetes initiatives impacting on the study were outlined in the study proposal and are monitored by the study now. Monitoring occurs through policies and procedures to identify potential roadblocks in the form of risk registries which are managed by the MDPS study board and implementation team.

However, the nature of implementing prevention programs in the real world means that some of the roadblocks could not be predicted at any early stage and the study team had to deal with issues as they arose. Furthermore, once the Life! program was implemented as part of government policy, time pressures and program momentum impacted on the ability to do a detailed risk analysis.

#### Do the roadblocks identified relate to what others have found?

The issues encountered when implementing the MDPS are similar to those outlined by leaders in population level diabetes prevention initiatives [23,24]. They argue that in order for successful execution and sustainability of large population wide diabetes prevention programs consideration needs to be given to factors such as cost and resources, expertise of the research and implementation team, effective participant screening and uptake of the intervention, maintained investment from funding sources, ensuring management systems are of high quality and results are disseminated and utilised in an efficient way to all levels of government, the wider community and individuals.

## Summary

The experiences of conducting such a novel trial as the MDPS have been invaluable. Table 1 provides a full list of barriers encountered in the implementation of the MDPS and proposed solutions. The lessons learnt and knowledge gained will inform the future execution of this trial in the coming years and enhance its potential to contribute to the ongoing Life! program. We anticipate that these results will also be beneficial to other researchers conducting similar trials in the public health field. Like Vedelø (2011), we also recommend that researchers openly share their experiences, barriers and challenges when conducting RCTs and implementation research [44]. We encourage them to describe the factors that may have inhibited or enhanced the desired outcomes so that the academic community can learn and expand the research foundation of implementation salvage.

## Competing interests

The authors declare that they have no competing interests.

## Authors’ contribution

JD, EJ and RC and were responsible for the research question, design of the study, and for obtaining funding. AH and NDL wrote the first draft of this manuscript and were responsible for the revisions, along with EJ and JD. DA, SO, AT, ES, CB and GJ contributed to specific sections of the manuscript. RC is the team's expert in economic evaluations and was involved in the design of the study. JD is the general supervisor of the study and was involved in revising the article. All authors read and approved the final version of the manuscript.

## Pre-publication history

The pre-publication history for this paper can be accessed here:

http://www.biomedcentral.com/1471-2458/12/806/prepub
